# Dyslexia associated gene *KIAA0319* regulates cell cycle during human neuroepithelial cell development

**DOI:** 10.3389/fcell.2022.967147

**Published:** 2022-08-09

**Authors:** Steven Paniagua, Bilal Cakir, Yue Hu, Ferdi Ridvan Kiral, Yoshiaki Tanaka, Yangfei Xiang, Benjamin Patterson, Jeffrey R. Gruen, In-Hyun Park

**Affiliations:** ^1^ Department of Genetics, Yale Stem Cell Center, Yale School of Medicine, New Haven, CT, United States; ^2^ Department of Biostatistics, Yale School of Public Health, New Haven, CT, United States; ^3^ Department of Medicine, Maisonneuve-Rosemont Hospital Research Centre, University of Montreal, Montreal, QC, Canada; ^4^ School of Life Science and Technology, ShanghaiTech University, Shanghai, China; ^5^ Departments of Pediatrics and of Genetics, Yale School of Medicine, New Haven, CT, United States

**Keywords:** KIAA0319, cell cycle, hESCs, NPCs, differentiation

## Abstract

Dyslexia, also known as reading disability, is defined as difficulty processing written language in individuals with normal intellectual capacity and educational opportunity. The prevalence of dyslexia is between 5 and 17%, and the heritability ranges from 44 to 75%. Genetic linkage analysis and association studies have identified several genes and regulatory elements linked to dyslexia and reading ability. However, their functions and molecular mechanisms are not well understood. Prominent among these is *KIAA0319*, encoded in the DYX2 locus of human chromosome 6p22. The association of *KIAA0319* with reading performance has been replicated in independent studies and different languages. Rodent models suggest that *kiaa0319* is involved in neuronal migration, but its role throughout the cortical development is largely unknown. In order to define the function of *KIAA0319* in human cortical development, we applied the neural developmental model of a human embryonic stem cell. We knocked down *KIAA0319* expression in hESCs and performed the cortical neuroectodermal differentiation. We found that neuroepithelial cell differentiation is one of the first stages of hESC differentiation that are affected by *KIAA0319* knocked down could affect radial migration and thus differentiation into diverse neural populations at the cortical layers.

## Introduction

Reading is a complex trait influenced by both environmental and genetic factors. Dyslexia, also known as reading disability, is characterized by reading difficulty in the setting of normal intelligence and adequate education. Dyslexia is associated with long-term detrimental effects on educational achievement and socioeconomic status (Schatschneider and Torgesen, 2004). It is the most common learning disability, with a prevalence of 5–17% and a heritability of 0.34–0.76 (DeFries et al., 1987; Shaywitz and Shaywitz, 2005; Hawke et al., 2006). Genetic studies have identified nine dyslexia loci (DYX1-9), contingent on aspects of reading performance (Gayan et al., 1999; Fisher and DeFries, 2002). The most frequently replicated locus is DYX2 on chromosome 6p21.3, and the most frequently replicated dyslexia genes on DYX2 are *KIAA0319* and *DCDC2* (Deffenbacher et al., 2004; Francks et al., 2004; Meng et al., 2005; Brkanac et al., 2007; Zhao et al., 2016).

Animal models show that kiaa319 interacts with intracellular trafficking protein AP-2. Additionally, KIAA0319 undergoes N- and O- glycosylation, associated with the plasma membrane, suggesting that KIAA0319 plays a role in cell maintenance and cell-cell signaling in the endocytosis pathway (Velayos-Baeza et al., 2008; Levecque et al., 2009). Kiaa0319 is also associated with extracellular signaling pathways (Franquinho et al., 2017; Wu et al., 2020), via Smad2/3. Both endocytosis and extracellular signaling pathways are essential for regulating neurogenesis (Cope and Gould, 2019).

Several dyslexia genes, including kiaa0319 and dcdc2, affect neuronal radial migration during rat embryogenesis (Peschansky et al., 2010). Radial migration begins at the neuroepithelial cell stage of neurogenesis. Individual cortical layers are then populated by neuronal progenitor cells (Stockinger et al., 2011; Ji et al., 2017). Neuronal progenitor cells are differentiated into neurons, astrocytes, and oligodendrocytes, providing the diversity in neuronal subtypes essential for neural circuitry construction, maintenance, and function (Gotz and Huttner, 2005; Klausberger and Somogyi, 2008; Bergles and Richardson, 2015; Molofsky and Deneen, 2015; Durkee and Araque, 2019). Regulation of neuronal progenitor cell differentiation in the dentate gyrus of the hippocampus is associated with learning and memory and critical for propagating new neurons in the adult brain (Cameron and Glover, 2015).

While animal models provide insights into some of the cellular functions of dyslexia genes (Paracchini et al., 2006; Szalkowski et al., 2012), they do not account for the complexity of reading or the critical skills that subserve reading, such as decoding, phonological awareness, or orthographic coding. Modeling genetic variants associated with specific human neurons and tissues could provide a way to reference findings to brain regions critical to reading (Powers et al., 2016; Fitch, 2017; Kirby, 2017).

Gene editing approaches in pluripotent cells, such as human embryonic stem cells (hESCs) and induced pluripotent stem cells (iPSCs), have been successfully used to model the effects of genetic variants on neuronal differentiation (Wang et al., 2017; Xiang et al., 2020). Pluripotent stem cells can be induced to differentiate into many cell types, including glutamatergic neurons, GABAergic neurons, and glial cells that populate the human brain (Damdimopoulou et al., 2016). Neuroectodermal differentiation of hESCs can reproduce developmental stages during embryonic brain development and gene expression patterns (Howard et al., 2008). Combined with gene editing tools, hESCs provide powerful experimental systems for dissecting the function of genes associated with neurodevelopmental or neuropsychiatric disorders (Kolagar et al., 2020).

The *KIAA0319* variants associated with dyslexia are at the promoter region and thus the cell type and the temporal regulation of *KIAA0319* expression is perhaps a major cause of the dyslexia. Here, as one of the first attempts to understand how *KIAA0319* regulates the human cortical development, we set out to investigate the function of *KIAA0319* in the context of the loss of function. We used a hESC model of cortical neural differentiation and probe the function of *KIAA0319* during neurogenesis. We knocked down *KIAA0319* in hESCs and performed neuroectodermal differentiation to examine how the neural differentiation is affected by loss of function of *KIAA0319* throughout the neuronal development. We found that KIAA0319 is critical in neuroepithelial cell differentiation, which could affect radial migration and further differentiation into diverse populations of brain cells. These results suggest that one of the mechanisms whereby dysregulated expression of *KIAA0319* could influence human cortical neurodevelopmental process is the neuroepithelial differentiation, which could underlie the complex traits such as reading.

## Results

### *KIAA0319* regulates the transition of neuroepithelial cells into neuronal progenitor cells

To determine how *KIAA0319* gene expression changes, we first examined transcriptome profiles of somatic tissues during human fetal development. *KIAA0319* is strongly expressed throughout human cortical development and in all brain regions (Supplementary Figures S1A–D), (Pletikos et al., 2014; Cardoso-Moreira et al., 2019).

**FIGURE 1:**
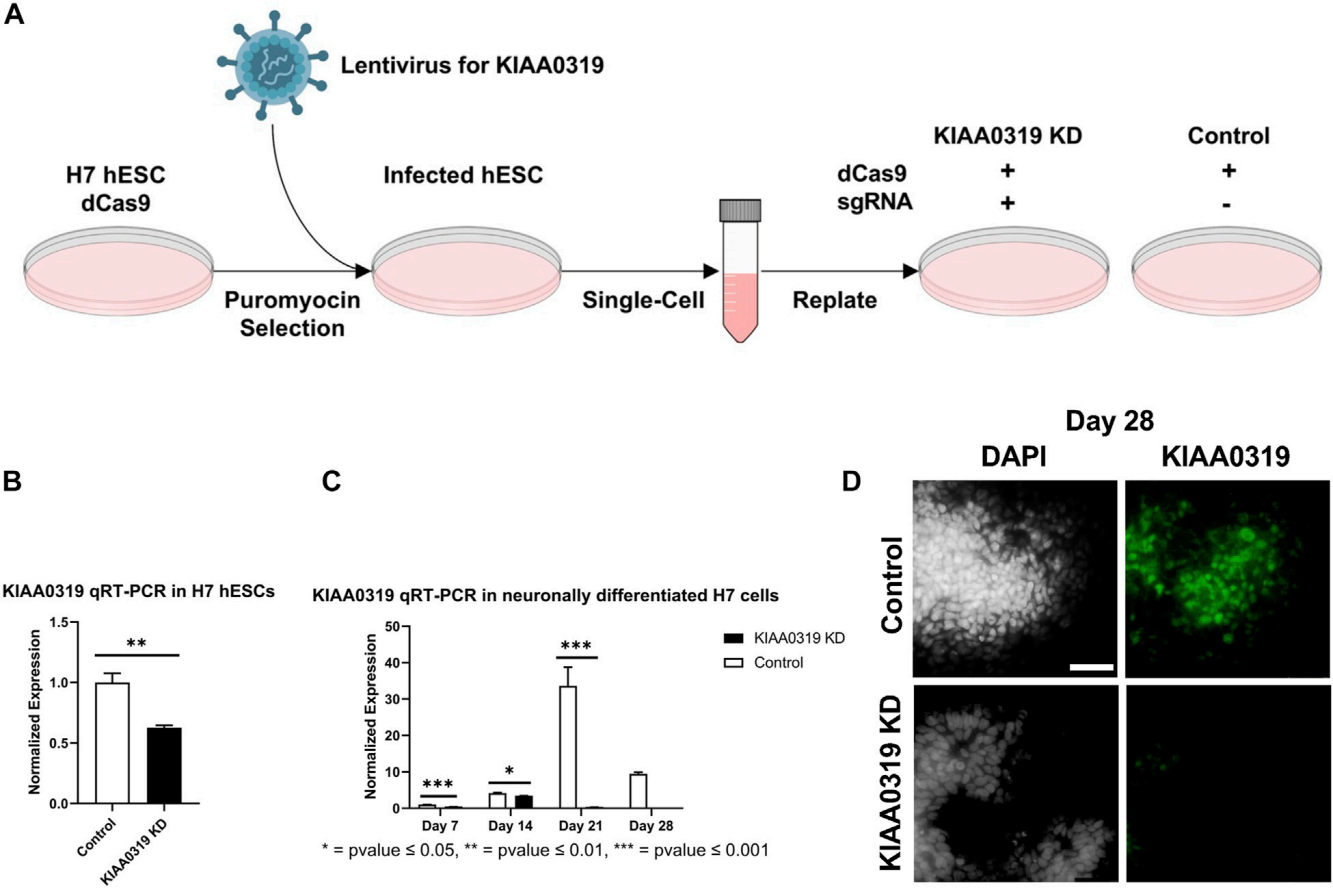
Depletion of *KIAA0319* by CRISPR interference (CRISPRi) **(A)** Workflow for generating KIAA0319 KD using CRISPRi in hESCs. **(B)** Confirmation of *KIAA0319* KD by qRT-PCR in H7 cells after transfection and puromycin selection. **(C)** Confirmation of *KIAA0319* KD by qRT-PCR on days 7, 14, 21, and 28 of neuronal differentiation in H7 cells by qRT-PCR in neuronally differentiated H7 cells. **(D)** Immunofluorescence images of *KIAA0319* KD and control on day 28. Green: KIAA0319, grey: DAPI. Scale bar: 50 µm.

**FIGURE 2:**
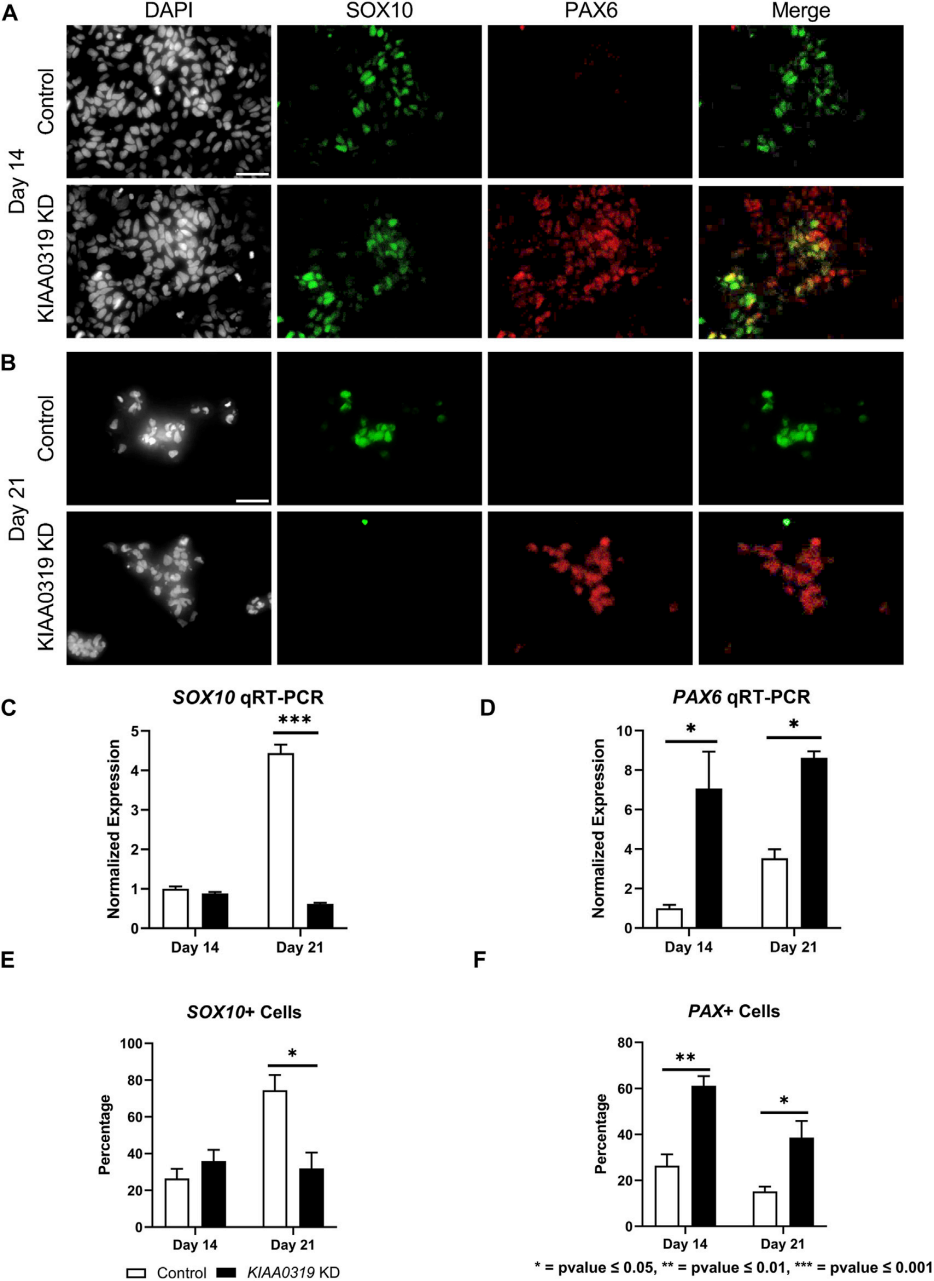
*KIAA0319* KD decreases expression of neuronal epithelial marker SOX10 but increases neuronal progenitor marker PAX6. **(A)** After 14 days of neuronal differentiation, immunofluorescence was performed for SOX10 (green), PAX6 (red), and DAPI (grey). **(B)** After 21 days of neuronal differentiation, immunofluorescence was performed for SOX10 (green), PAX6 (red), and DAPI (grey). qRT-PCR for SOX10 **(C)** and PAX 6 **(D)** were performed on days 14 and 21 of *KIAA0319* KD and Control H7 cells. The percentage of the population of SOX10+ **(E)** or PAX6+ **(F)** cells were calculated on day 14 and day 21 of differentiation for *KIAA0319* KD and Control H7 cells.

To study the potential effects of KIAA0319 on neurogenesis, we knocked down (KD) expression using CRISPRi/dCas9-KRAB in H7^dCas9−KRAB-b6^ hESCs (Figure 1A) targetting the transcription start site. Both qRT-PCR and immunofluorescence confirmed decreased expression of *KIAA0319* in CRISPRi cells compared to controls during neuronal differentiation at days 7, 14, 21, and 28 (Figures 1B–D). We then assessed the expression of genes representative of transitional stages during neuronal differentiation by qRT-PCR and immunostaining on days 7, 14, 21, and 28 of cortical differentiation. We focused on neuronal stage lineage markers *SOX10*, *PAX6*, *TBR2*, *TBR1*, and *MAP2* to quantitate the population of neuroepithelial, neuronal progenitor, immature neuron, and mature neurons, respectively. Compared to controls, SOX10 expression by qRT-PCR and immunofluorescence, a marker of neuroepithelial cells, was suppressed at day 21 in *KIAA0319* KD cells (Figures 2A–C,E). Conversely, PAX6 expression by qRT-PCR and immunofluorescence, a marker of neuronal progenitor cells, increased in *KIAA0319* KD cells on days 14 and 21 (Figures 2D–F). Together, these results suggest that *KIAA0319* is critical for regulating the transition of SOX10 + neuroepithelial cells to PAX6+ neuronal progenitor cells.

### *KIAA0319* KD arrests cells in a non-proliferative neuronal progenitor cell stage

**FIGURE 3:**
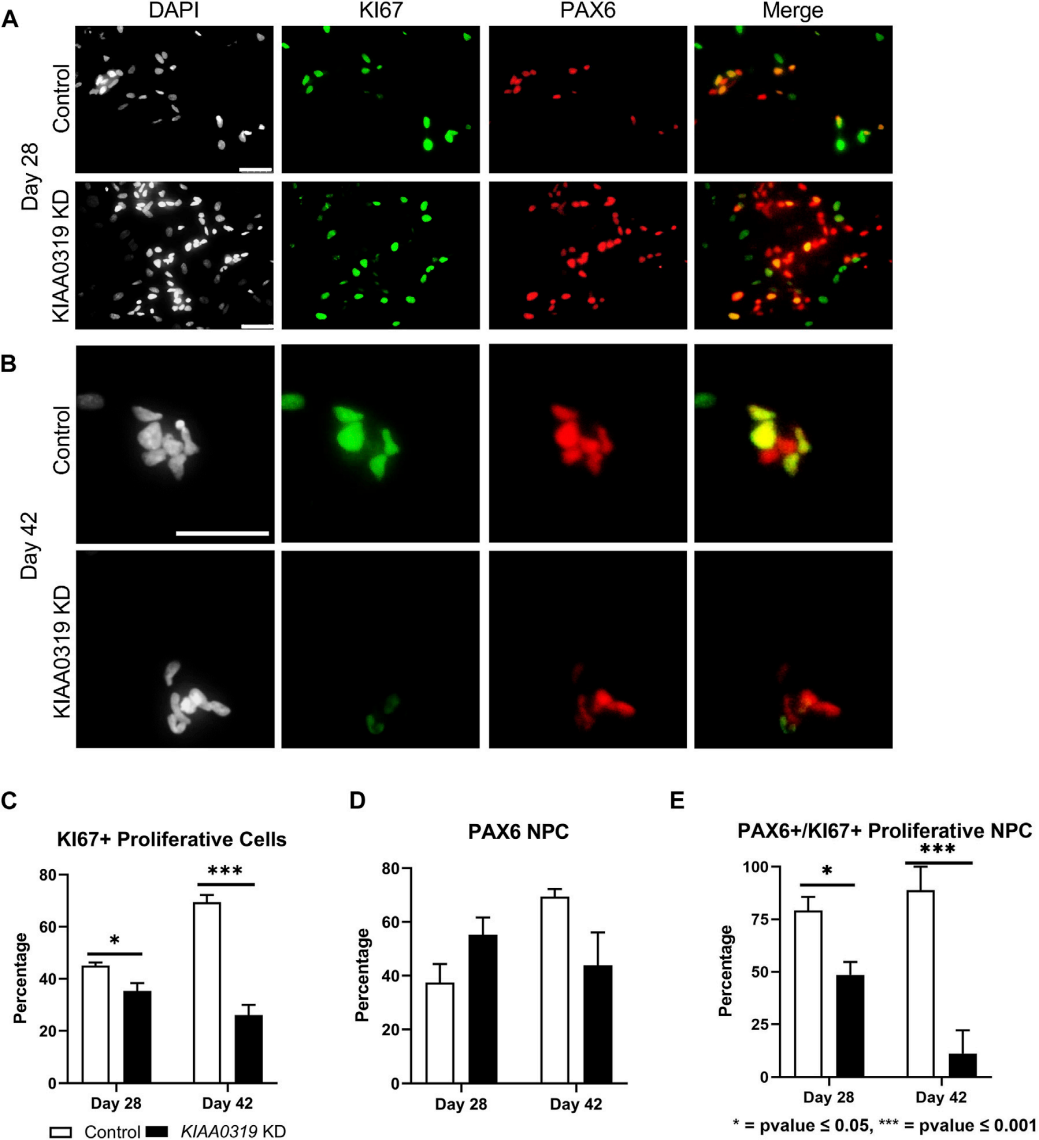
*KIAA0319* KD decreases the percentage of proliferating neuronal progenitor cells. **(A–B)** Immunostaining of KIAA0319 KD and control cells for KI67 (green) and PAX6 (red) in Dapi (gray) on day 28 **(A)** and day 42 **(B)** of neuronal differentiation. Scale bar: 50 µm. Percentage of KI67 + cells **(C)** and PAX6+ population **(D)** among DAPI + cells at day 28 and day 42. Percentage of PAX6+ cells among KI67 + population at day 28 and day 42 **(E)**.

Next, we characterized how *KIAA0319* regulates PAX6+ neuronal progenitor cells at later stages of differentiation. We simultaneously measured KI67, a marker for proliferation, and PAX6 by qRT-PCR and immunofluorescence, and quantified the proliferation of neuronal progenitor cells at two later time points of differentiation (days 28 and 42) (Figures 3A,B). We found that the fraction of KI67 + cells remained relatively low in *KIAA0319* KD cells compared to controls (Figure 3C), while the fraction of PAX6+ cells was relatively the same (Figure 3D). However, the fraction of KI67 + cells among PAX6+ cells decreased, suggesting an overall decrease in the population of proliferating neuronal progenitor cells (Figure 3E). Overall, these results suggest that *KIAA0319* KD could be driving PAX6+ neuronal progenitor cells into cell cycle arrest, inducing apoptosis in the proliferating cells, or prematurely inducing transition into downstream stages of neuronal differentiation.

**FIGURE 4:**
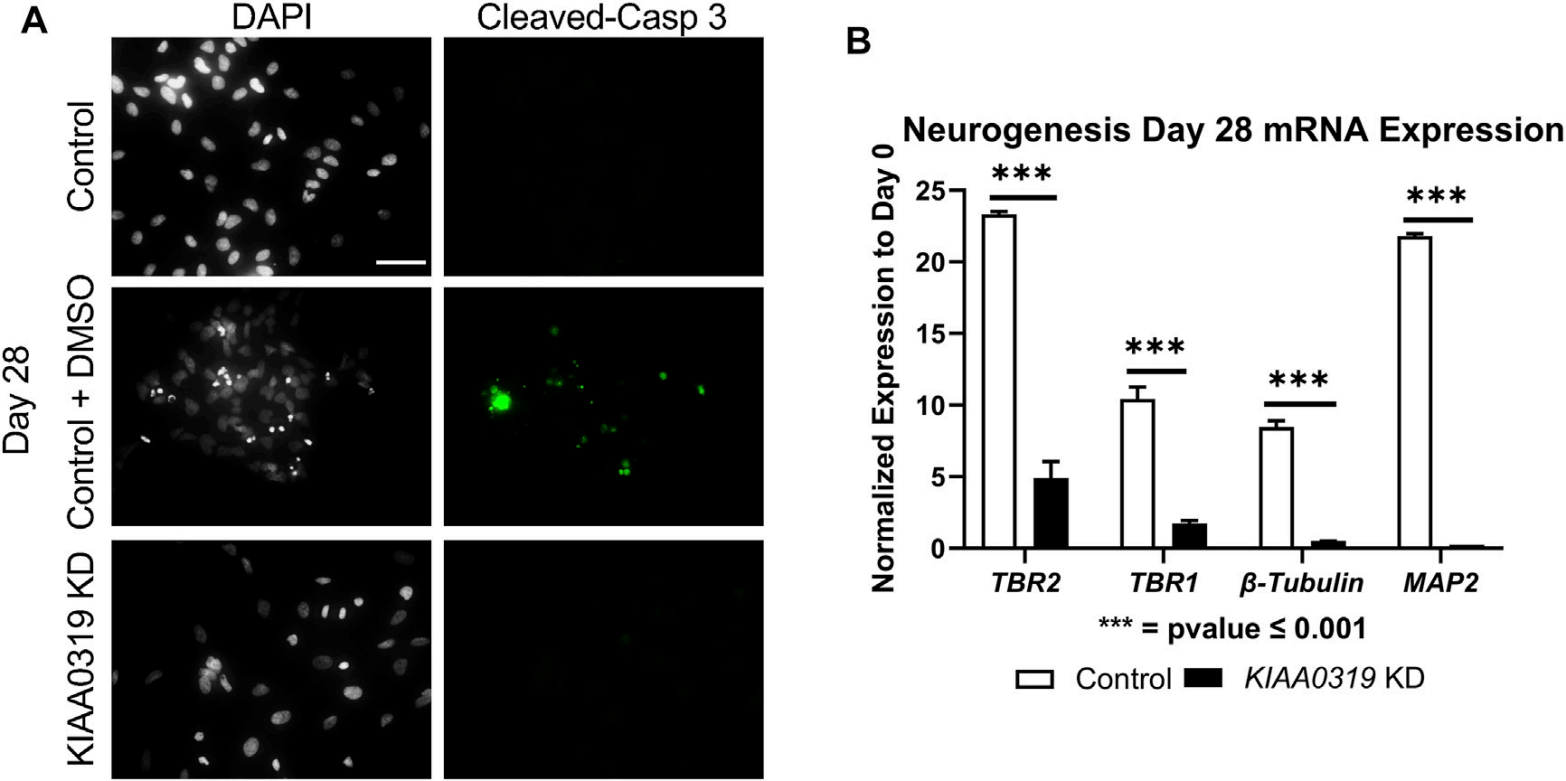
*KIAA0319* KD cells do not continue differentiating. **(A)** Images of *KIAA0319* KD, Control, Control + DMSO (Positive Apoptosis Control) at day 28 of neuronal differentiation stained with cleaved-caspase 3 (green) and DAPI (grey). **(B)** QRT-PCR at day 28 for neuronal lineage markers *TBR1, TBR2, β-Tubulin,* and *MAP2.*

To assess whether the decrease in KI67 + PAX6+ neuronal progenitor cells was due to apoptosis, we stained KD and control cells with cleaved caspase-3 antibody, a marker of cell death induction, at day 28 (Figure 4A). *KIAA0319* KD did not induce apoptosis, suggesting that the decrease in KI67 + PAX6+ neuronal progenitor cells was not due to dying cells. Next, we examined markers at later stages of neuronal differentiation to quantify if *KIAA0319* KD induces the premature transition from neuronal progenitor cells into downstream stages of neuronal differentiation. Compared to controls at day 28, *KIAA0319* KD significantly decreased the expression of markers of intermediate progenitor cells (TBR2), immature neuronal cells (TBR1 and *β-Tubulin*), and mature neuronal cells (MAP2) (Figure 4B). The results of these experiments show that *KIAA0319* KD arrests cells in a non-proliferative neuronal progenitor cell stage, and that *KIAA0319* is important for transitioning neuronal epithelial cells into and through the neuronal progenitor cell stage and to later stages of neuronal differentiation.

### *KIAA0319* KD drives neuronal progenitor cells into cell cycle arrest

**FIGURE 5:**
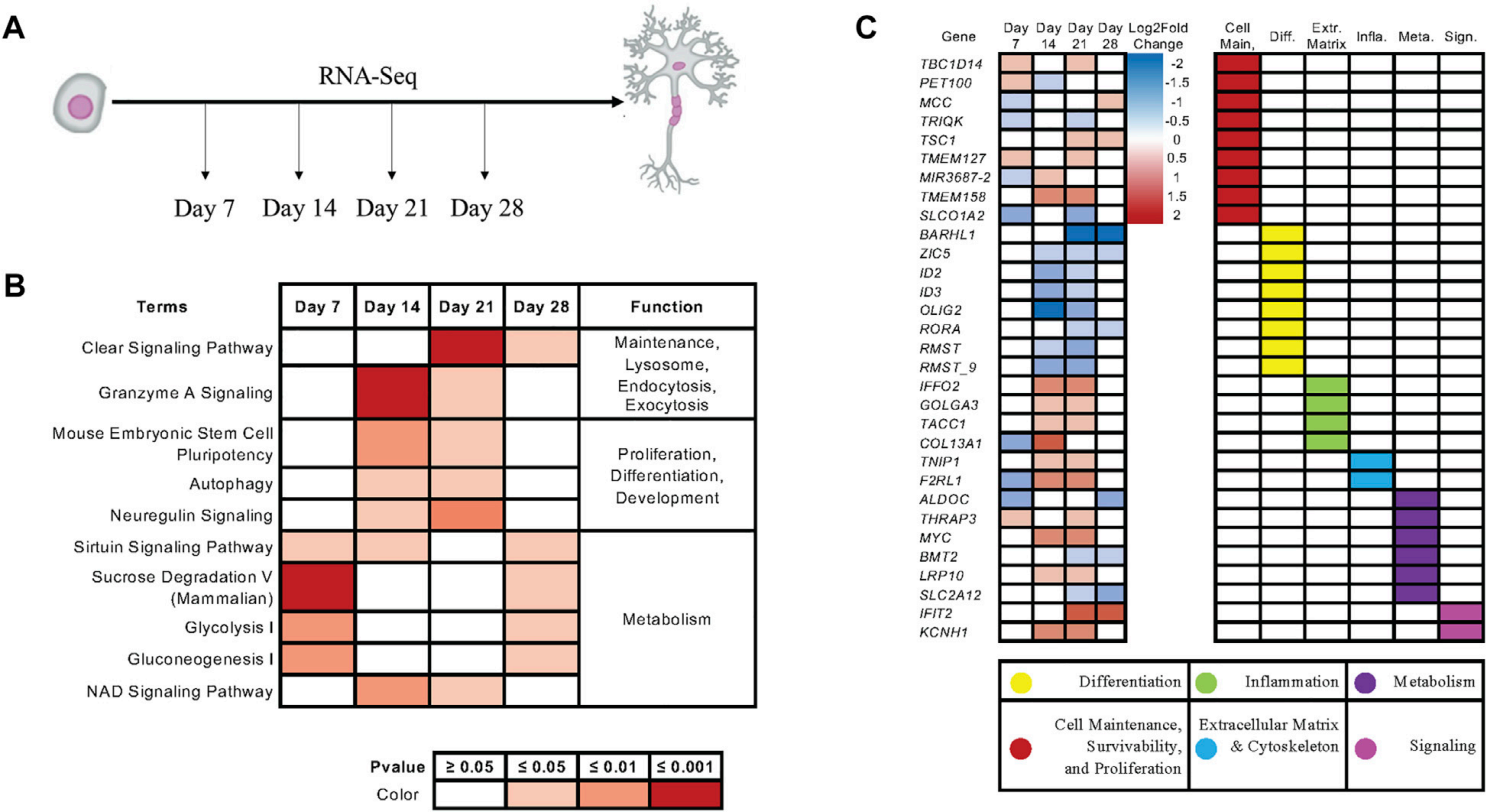
*KIAA0319* KD RNA-Seq shows pathways that promote cell cycle arrest of neuronal progenitor cells. **(A)** RNA Sequencing performed at four distinct timepoints separated by 7-day intervals during cortical neuronal differentiation. **(B)** Ingenuity Pathway Analysis revealed enrichment of pathways appearing in multiple time points. **(C)** Ingenuity Pathway Analysis revealed enrichment of genes appearing in multiple time points.

To examine how *KIAA0319* regulates proliferation in neuronal progenitor cells, we performed RNA-sequencing on the differentiating cells from day 7, 14, 21, and 28 (Figure 5A). Ingenuity Pathway Analysis (IPA) showed that endocytosis, exocytosis, lysosome, differentiation, proliferation, and metabolism are affected by the *KIAA0319* KD (Figure 5B), which were previously implicated in cell-cycle arrest of neuronal progenitor cells (Kobayashi et al., 2019; Harada et al., 2021; Zhang et al., 2021). Individual gene analysis showed a consistent decrease in expression of genes associated with differentiation at neuroepithelial and neuronal progenitor cell stages (Figure 5C). The changes in expression of genes associated with extracellular matrix, metabolism, cell maintenance, and cell-cell signaling further support the findings that *KIAA0319* KD drives neuronal progenitors into cell cycle arrest.

## Discussion

Here, we tested our hypothesis that the clinical association of *KIAA0319* with reading performance and dyslexia is a consequence of altered expression during early stages of neurogenesis. *KIAA0319* KD showed a decrease in the SOX10 + neuroepithelial cells concurrent with an increase in the PAX6 neuronal progenitors and suppression of ensuing neurogenesis. These findings are consistent with a decreased proliferative state at the neuronal progenitor cells due to cell cycle arrest, highlighting the importance of tight regulation of *KIAA0319* expression during a critical period of brain development.

Decreased expression of *KIAA0319* appears to promote early exit from the neuroepithelial cell stage and entry into the neuronal progenitor cell stage. This suggests that KIAA0319 plays a critical role as an upstream gatekeeper for neurogenesis, slowing or enhancing downstream differentiation by arresting cells at the neuronal progenitor cell stage. Transcriptome analysis suggests that *KIAA0319* could regulate cell proliferation through endocytosis-related pathways. Indeed, dysregulation of the endocytosis pathways has been shown to drive neuronal progenitor cells into a stage of cell cycle arrest (Zhang et al., 2021). Our results provide a new avenue for studying the regulation of neurogenesis in human neurons.

This is the first study to suggest that *KIAA0319*, which has been associated with reading performance in multiple independent studies, may act by regulating neurogenesis at a critical checkpoint between neuroepithelial cells and proliferating active neuronal progenitor cells. The population of mature adult neurons is tightly regulated by the proliferation of active neuronal progenitors. Our results suggest that KIAA0319 is a critical check on this transition by either actively driving neuronal progenitors into cell cycle arrest, or by blocking release from cell cycle arrest into proliferating active cells and development into downstream lineages.

The *KIAA0319* genetic variants associated with reading and dyslexia in clinical studies are in the promoter region and regulate expression. Spatial and temporal regulation of *KIAA0319* expression that are affected by the variants could affect an isolated high-order trait such as reading without global effects on brain development or cognition; children and adults with dyslexia typically have normal brain development and cognition. While this study has a limitation in its design to reveal the function of the *KIAA0319* variants in human cortical neurogenesis, it is one of the first studies to show the role of *KIAA0319* in early neurogenesis in human cells. Future studies will be needed to define the function of the specific *KIAA0319* genetic variants associated with dyslexia in the genesis of specific cell lineages and brain development. Use of the 3D cell culture brain organoid models and single cell-based assay, like scRNA-seq, or scATAC-seq will be critical in reveaingl the cell type specific function of the variants.

## Methods

### Cell culture

HEK 293T cells were maintained in 6-well plates at 37°C with 5%CO_2_. Cells were tested for *mycoplasma* and passaged every 3 days using Gibco TrypLE™ Express with Phenol Red for dissociation. Culture media consisted of 88% v/v DMEM, 10% v/v FBS, 1% v/v Glutamax, and 1% v/v Penicillin/Streptomycin. H7^dCas9−KRAB-b6^ (WiCell, WA07, H7) human ESCs were maintained in feeder-free culture conditions using Matrigel-coated cell culture dishes and mTeSR1 media at 37°C and 5% CO_2_. Cells were tested for *mycoplasma* and passaged every 7 days following dissociation with Dispase (0.83 U/ml). All experiments involving hESCs were approved by the Yale Embryonic Stem Cell Research Oversight Committee (ESCRO).

### Neuronal differentiation

Pre-differentiated cells were removed before hESC colonies, dissociated into single cells using Accutase, and plated on 24-well plates at 500,000 cells/well with 50 µM Y27632 and mTeSR1. Once plates reached 90% confluency, cells were grown for 10 days with neural induction media (47.5% v/v; DMEM/F12, 47.5% v/v Neurobasal media, 2% v/v B27 supplement, 1% v/v DMEM-NEAA, 1% v/v N2 supplement, 1% v/v Glutamax, 20 μg/ml Insulin, 100 nM LDN-193189, 50 µM β-Mercaptoethanol, 10 μM SB431542, 2 µM XAV939), changing media every day. Cells were dissociated using Accutase on day 11 and plated on new 24-well plates along with 50 µM Y27632 and progenitor expansion media (neural induction media without inhibitors: LDN-193189, SB431542, and XAV939), changing media every other day. On day 19, culture media was changed to maturation media (95% v/v Neurobasal media, 2% v/v B27 supplement, 1% v/v N2 Supplement, 1% v/v Glutamax, 1% v/v Penicillin/Streptomycin) with 25 ng/ml BDNF, changing media every 2 days for 2 weeks and every 4 days thereafter. Cells were dissociated using Accutase and replated on new 24-well plate on day 25 at 100,000 cells/well when confluency was reached.

### *KIAA0319* lentivirus

Single Guide RNA (sgRNA) and reverse complement were designed using CRISPR PAM sites (crispr.mit.edu) targeting the transcription start site of *KIAA0319* (*KIAA0319* KD plasmid) and synthesized by Keck (sgRNA *KIAA0319* KD and Control listed in Table 1). Oligos were annealed using 50% v/v NEB Annealing Buffer, 46% v/v ddH_2_0, 2% v/v top oligo, and 2% v/v bottom oligo (Oligos listed in Table 1). Oligos were incubated at 95 °C and left to cool to room temperature. Plasmid pCRISPia-V2, encoding a Puromycin resistance gene, had a sgRNA insertion site opened using digestion enzymes BstXI and Blpl. Ligation reactions consisted of 70% v/v ddH_2_O, 10% v/v of 10X T4 ligase buffer, 10% v/v 1:20 diluted annealed oligos, 5% pCRISPia-V2 (totaling 100 ng of digested plasmid), and 5% v/v T4 Ligase at room temperature for 1 h.

**TABLE 1:** CRISPRi oligos.

Gene target	Forward sgRNA	Bottom sgRNA
*KIAA0319*	ttg​TGG​TAA​CCG​CGG​CGG​CGG​AAA​GGg​ttt​aag​agc	tta​gct​ctt​aaa​cCC​TTT​CCG​CCG​CCG​CGG​TTA​CCA​caa​caa​g
Control	ttg​GAC​CAG​GAT​GGG​CAC​CAC​CCg​ttt​aag​agc	tta​gct​ctt​aaa​cGG​GTG​GTG​CCC​ATC​CTG​GTc​caa​caa​g

Ligation reactions were transformed into One Shot™ TOP10 Chemically Competent cells following manufactures instructions and plated on 10 cm LB Agar + Ampicillin (100ug/ml) plates from Recombinant Technologies, LLC at 37°C for culture overnight. Surviving colonies were cultured overnight in Gibco LB Broth with Ampicillin at 100ug/ml in a 37°C shaker, followed by plasmid purification using QIAGEN Plasmid Mini Kit and sequence confirmation.

*KIAA0319* KD and Control plasmids were individually transfected into HEK293T cells along with 2.5 ugs of packaging plasmids using X-treme GENE nine DNA transfection reagent following manufacturer’s instructions. Supernatants containing lentivirus were collected and concentrated 48 h after transfection and stored at −80°C. H7 cells were infected with *KIAA0319* KD lentivirus and control lentivirus. Infected cultures were selected following puromycin selection for 5 days.

### Real time quantitative PCR

Four wells of a 24-well plate of H7 cells transfected with *KIAA0319* KD or control samples were collected on days 7, 14, 21, and 28 of neuronal differentiation per replicate. Samples were homogenized using QIAGEN QIAshredder columns, and total RNA was isolated using the RNeasy Mini Kit following the manufacturer’s instructions. One ug of total RNA was used to synthesize cDNA using amfiRivert cDNA Synthesis Platinum Master Mix. Real-time quantitative PCR (RT-PCR) was performed using amfiSure qGreen Q-PCR Master Mix (2X), Low Rox on a CFX96 R-PCR System at amfiSure manufacture cycling conditions with primers listed in Table 2. Gene expression was quantified using the ∆∆C_T_ method with *GAPDH* as a housekeeping gene control. *t*-test was performed for the statistical analysis. *p* values are given at each figure.

**TABLE 2:** qRT-PCR gene target primers.

Gene target	Forward primer	Reverse primer
*KIAA0319*	GGA​AAC​CAG​AGC​AGT​GAC​GAT​C	GAA​GGT​ATG​GCG​TCT​GTA​CTC​C
*NANOG*	CTC​CAA​CAT​CCT​GAA​CCT​CAG​C	CGT​CAC​ACC​ATT​GCT​ATT​CTT​CG
*SOX10*	ATG​AAC​GCC​TTC​ATG​GTG​TGG​G	CGC​TTG​TCA​CTT​TCG​TTC​AGC​AG
*PAX6*	CTG​AGG​AAT​CAG​AGA​AGA​CAG​GC	ATG​GAG​CCA​GAT​GTG​AAG​GAG​G
*TBR2*	AAA​TGG​GTG​ACC​TGT​GGC​AAA	CTC​CTG​TCT​CAT​CCA​GTG​GGA​A
*TBR1*	TCA​CTG​GAG​GTT​TCA​AGG​AGG​C	TTT​CTT​GGC​GCA​TCC​AGT​GAG​C
*MAP2*	CCA​CCT​GAG​ATT​AAG​GAT​CA	GGC​TTA​CTT​TGC​TTC​TCT​GA
*β-Tubulin*	TCA​GCG​TCT​ACT​ACA​ACG​AGG​C	GCC​TGA​AGA​GAT​GTC​CAA​AGG​C
*GAPDH*	GTC​TCC​TCT​GAC​TTC​AAC​AGC​G	ACC​ACC​CTG​TTG​CTG​TAG​CCA​A
*GFAP*	GTA​CCA​GGA​CCT​GCT​CAA​T	CAA​CTA​TCC​TGC​TTC​TGC​TC
*OLIG2*	ATG​CAC​GAC​CTC​AAC​ATC​GCC​A	ACC​AGT​CGC​TTC​ATC​TCC​TCC​A

### Immunostaining

Twelve wells of a 24-well plate of *KIAA0319* KD or Control cells were washed once in PBS before fixing in 4% formaldehyde/PBS at room temperature for 15 min and then washed in PBS (3 × for 15 min). In addition, samples were permeabilized by incubating with 0.1% Triton-100/PBS at room temperature for 1 hour and then washed in PBS (3 x for 15 min) before storing at 4°C in PBS.

Blocking was done with 3% BSA/0.1% Triton-100/PBS at 4°C for 2 hours before incubating with primary antibodies diluted to the manufacturer’s recommendation in 3% BSA/0.1% Triton-100/PBS at 4°C overnight. Samples were then washed in PBS (3 x for 15 min) before incubating with secondary antibody diluted in 3% BSA/0.1% Triton-100/PBS at room temperature for 1 h. Samples were washed in PBS (3 x for 15 min) before staining with DAPI to highlight nuclei for 5 minutes before final PBS wash. Immunofluorescence images were captured on a Leica TCS SP5 Spectral Confocal Microscope using Leica LAS AF software. Images were processed using ImageJ-Fiji.

### RNA-sequencing

Cell pellets from three biological replicates of *KIAA0319* KD and controls for days 7, 14, 21, and 28 were collected. RNA were isolated from cell pellets, processed for library prep, and then subjected to 150bp paired-end sequencing on a NOVA-seq for 50 million reads per sample at the Yale Center for Genomic Analysis (YCGA).

FASTQ files were processed using an RNA-Sequencing pipeline developed at the YCGA. Raw RNA-Sequencing reads were trimmed and aligned using Hierarchical Indexing for Spliced Alignment of Transcripts 2 (HISAT2) (Kim et al., 2019). Aligned reads are then concatenated using StringTie/Ballgown, followed by quality control using Picard (Frazee et al., 2015; Pertea et al., 2015). R package DESeq2 was used to generate summary reports and heatmaps using default setting (Love et al., 2014) (Supplementary Figures S2–S5).

## Ingenuity Pathway Analysis (QIAGEN)

Ingenuity Pathway Analysis (QIAGEN IPA, QIAGEN Inc, content version: 70750971) (Krämer et al., 2014) was used for pathway bioinformatics analysis. Significant genes that expressed differently in KD and control groups were identified by DESeq2 using Benjamini–Hochberg correction to guarantee a false discovery rate (FDR) ≤ 0.05. IPA performed a core analysis based on these significant genes and identified the significant pathways for each time point using a right-tailed Fisher’s exact test with a *p*-value threshold of 0.05. Finally, the pathways and genes were grouped by GO Term.

## Data Availability

The data presented in this study are deposited at the BioProject repository accession number PRJNA854587. The datasets presented in this study can be found in online repositories. The names of the repository/repositories and accession number(s) can be found in the article/Supplementary Material.
