# Supplementary material for: Dyslexia associated gene KIAA0319 regulates cell cycle during human neuroepithelial cell development
Source: Front Cell Dev Biol. 2022 Aug 9;10:967147. doi: 10.3389/fcell.2022.967147 (PMC9395643; doi:10.3389/fcell.2022.967147)

Supplemental Figure Legends

Supplemental Figure 1: (**A**) Bult Tissue Expression for *KIAA0319* provided by GTEx. Transcripts per million from all sampled brain regions in the GTEx project. (**B**) Bulk RNA-Sequencing provided by (Cardoso-Moreira et al., 2019) and selecting for *KIAA0319* expression. Expression data provided by (Pletikos et al., 2014) available from the Human Brain Transcriptome Database, (**C**) KIAA0319 signal intensity within the Neonatal Cortex. (**D**) *KIAA0319* signal intensity within the Brain Regions. cerebellar cortex (CBC), mediodorsal nucleus of the thalamus (MD), striatum (STR), amygdala (AMY), hippocampus (HIP) and 11 areas of neocortex (NCX): Frontal cortex (OFC, DFC, VFC, MFC, M1C), Parietal cortex (S1C, IPC), Temporal cortex (A1C, STC, ITC), Occipital cortex (V1C).

Supplemental Figure 2: Significant genes between KIAA0319 Knockdown and Control at day 7. (**A**) Heatmap for all 99 significant genes (**B**) Volcano plot for all significant genes determined by adjusted pvalue (**C**) Boxplot for top 30 significant genes. (**D**) Individual Boxplots for the top 30 significant genes.

Supplemental Figure 3: Significant genes between KIAA0319 Knockdown and Control at day 14. (**A**) Heatmap for all 85 significant genes (**B**) Volcano plot for all significant genes determined by adjusted pvalue (**C**) Boxplot for top 30 significant genes. (**D**) Individual Boxplots for the top 30 significant genes.

Supplemental Figure 4: Significant genes between KIAA0319 Knockdown and Control at day 21. (**A**) Heatmap for all 257 significant genes (**B**) Volcano plot for all significant genes determined by adjusted pvalue (**C**) Boxplot for top 30 significant genes. (**D**) Individual Boxplots for the top 30 significant genes.

Supplemental Figure 5: Significant genes between KIAA0319 Knockdown and Control at day 28. (**A**) Heatmap for all 89 significant genes (**B**) Volcano plot for all significant genes determined by adjusted pvalue (**C**) Boxplot for top 30 significant genes. (**D**) Individual Boxplots for the top 30 significant genes.

Suppl Figure 1

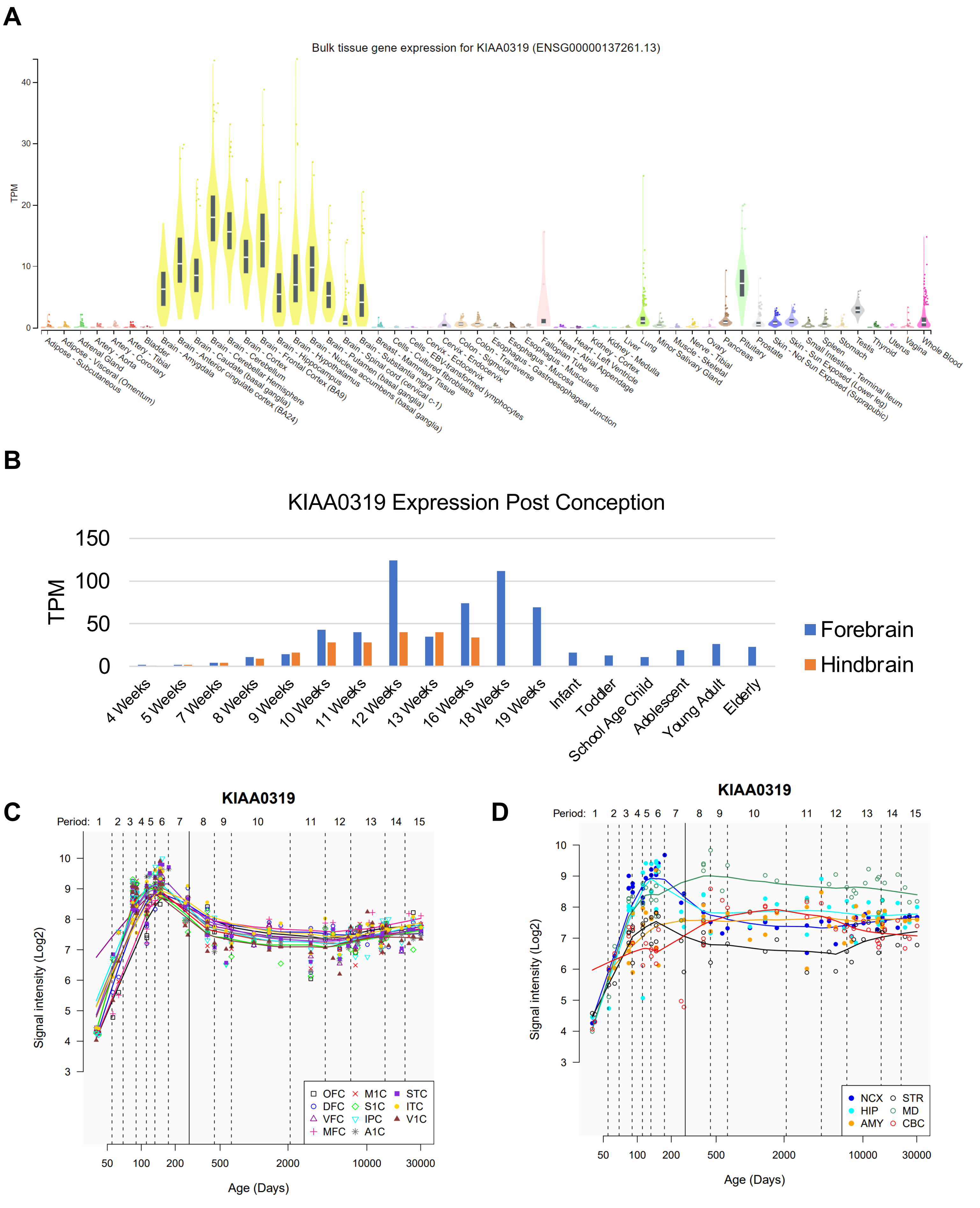

Suppl Figure 2

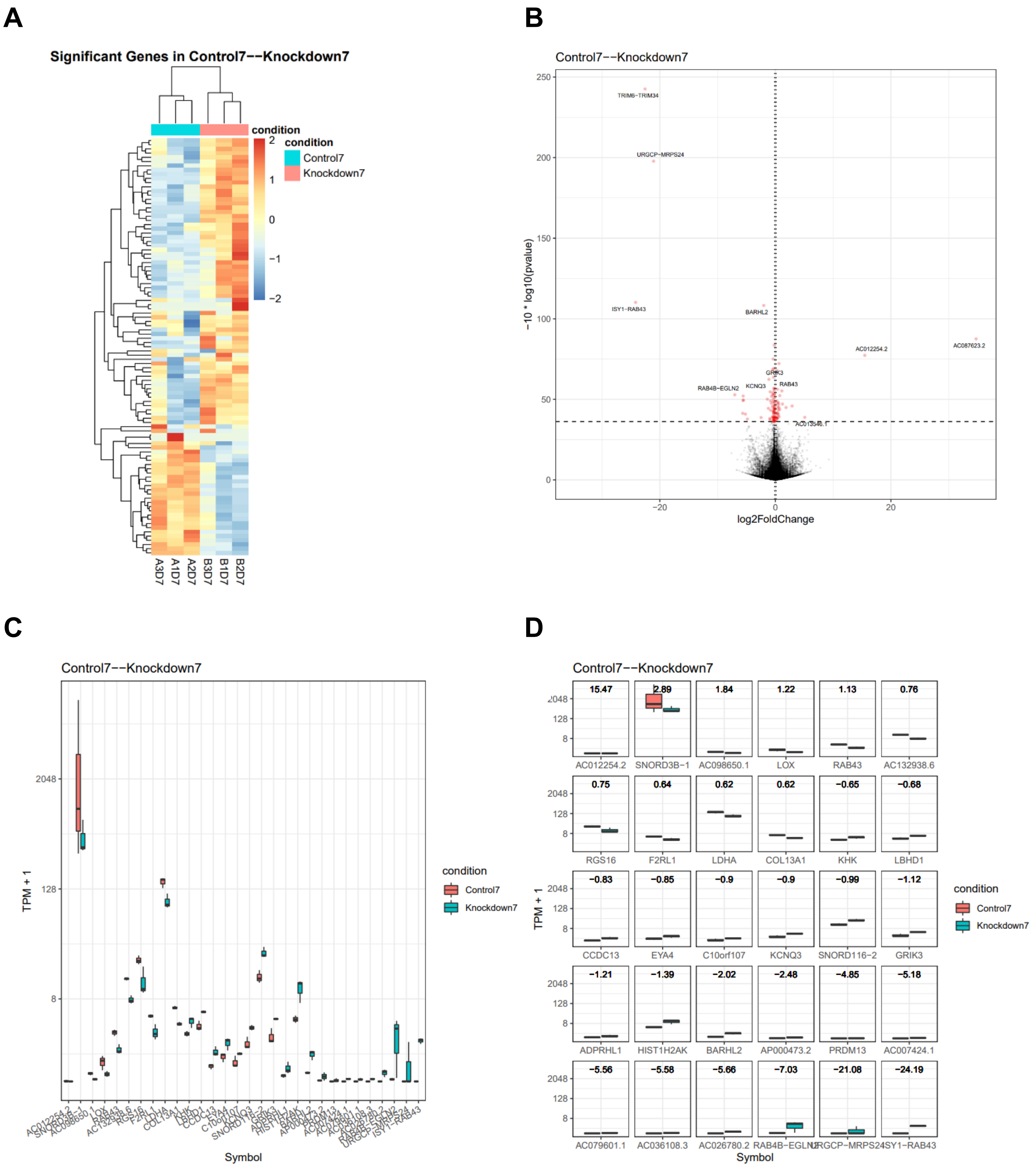

Suppl Figure 3

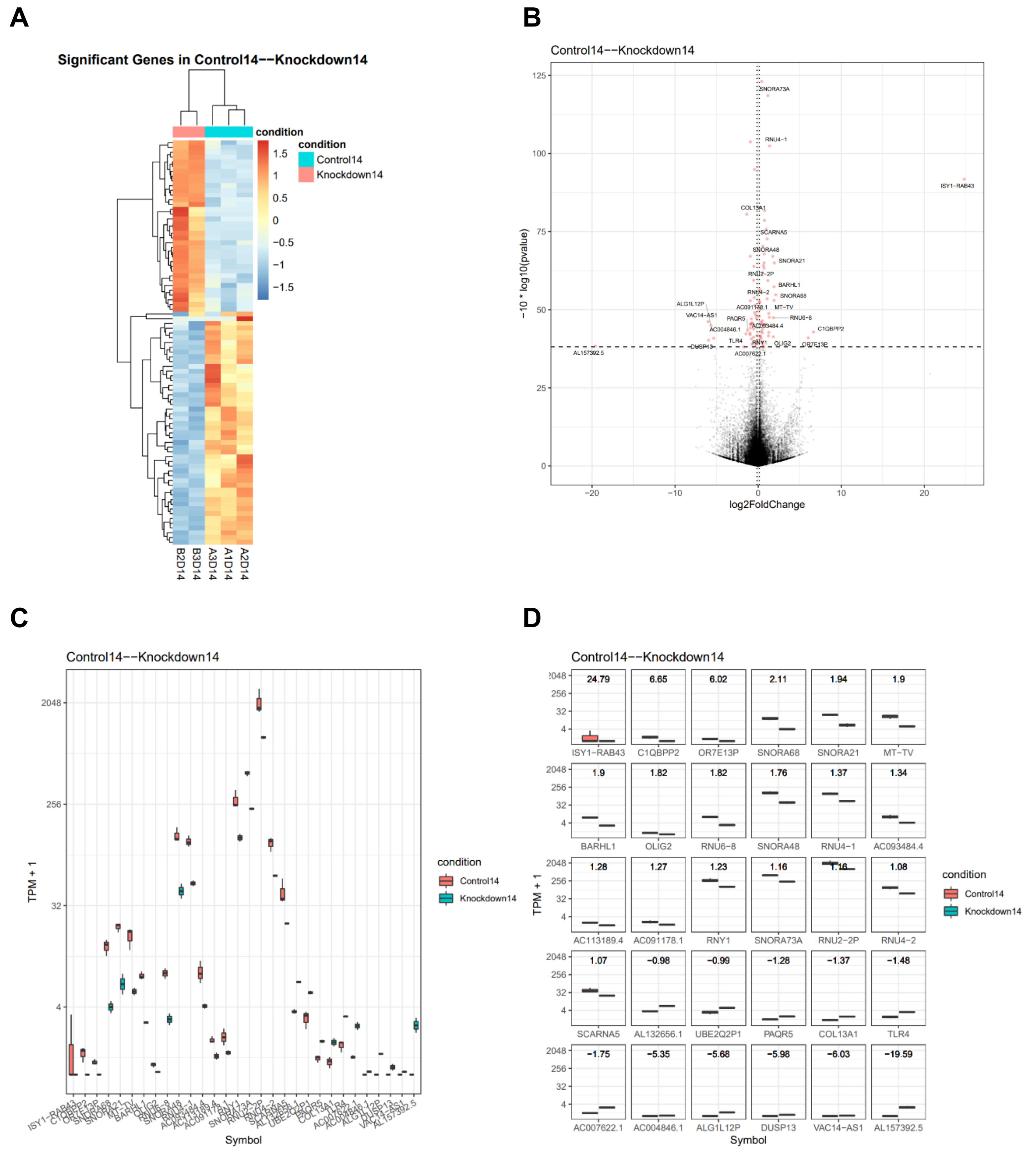

Suppl Figure 4

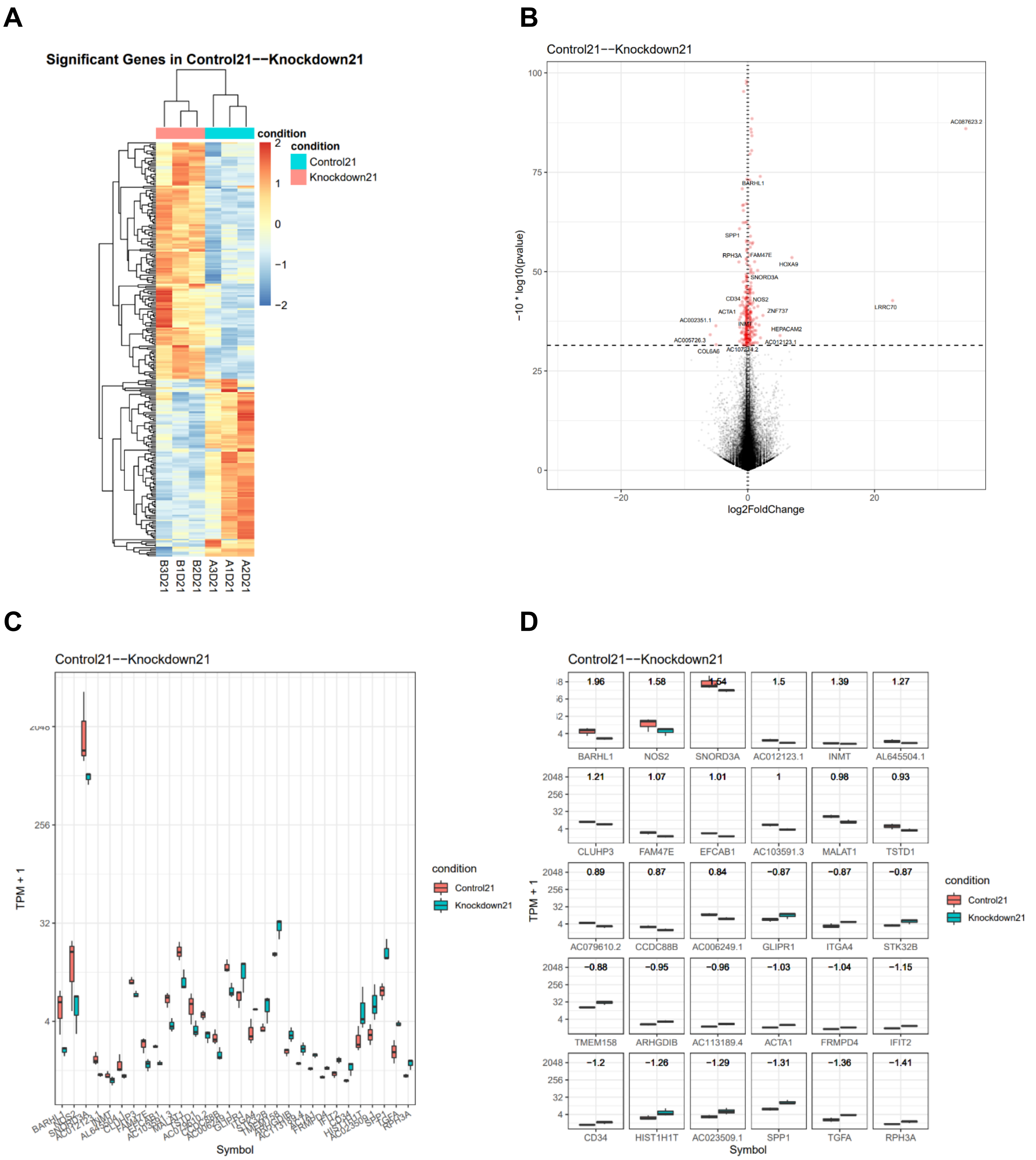

Suppl Figure 5

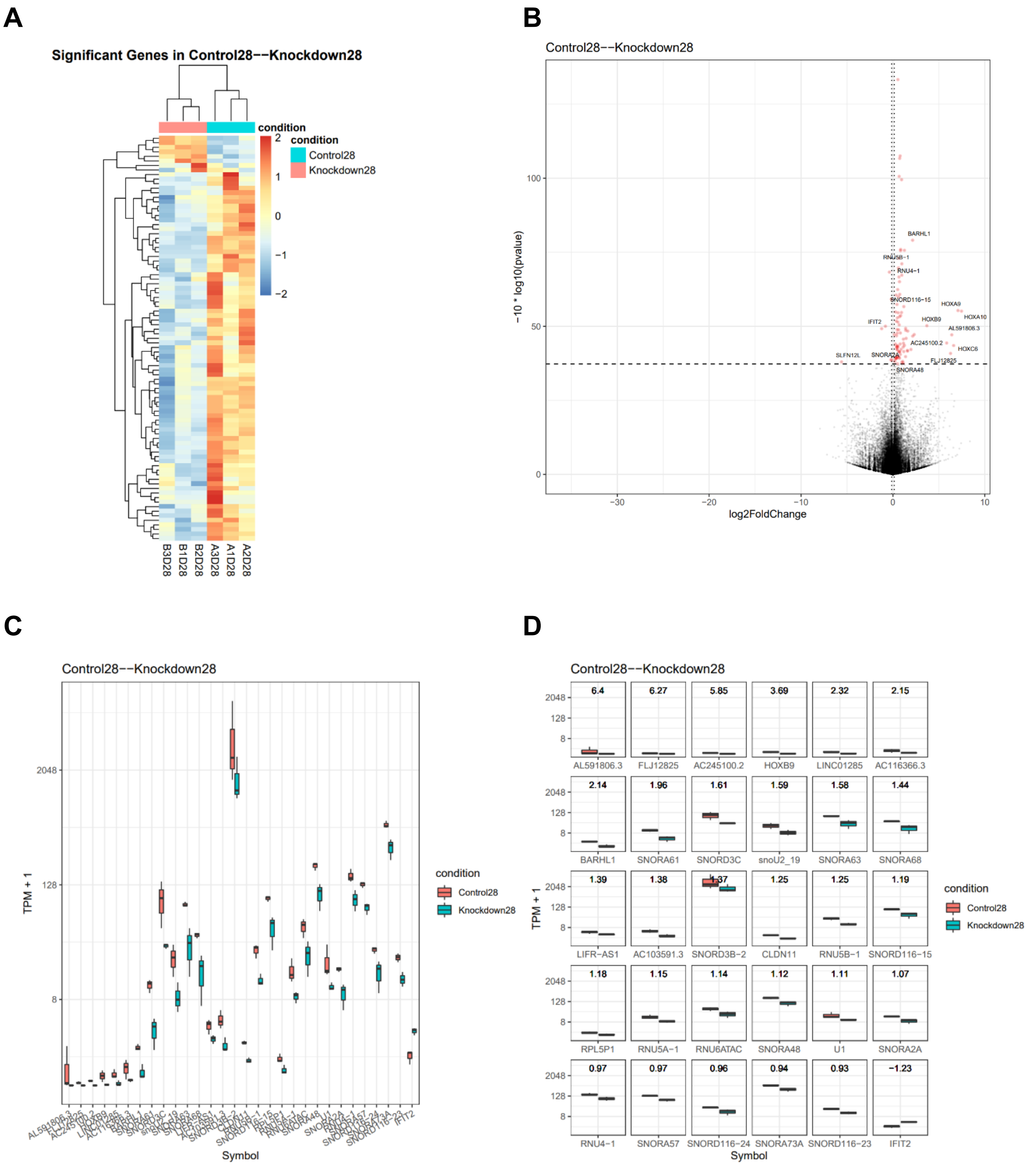

Supplement: Supplementary file 1 [file DataSheet1.PDF]
